# The impact of fetal gender on prematurity in dichorionic twin gestations after in vitro fertilization

**DOI:** 10.1186/1477-7827-8-57

**Published:** 2010-06-10

**Authors:** Andrea Weghofer, Katharina Klein, Maria Stammler-Safar, Christof Worda, David H Barad, Peter Husslein, Norbert Gleicher

**Affiliations:** 1 Department of Obstetrics & Gynecology, Medical University Vienna, Vienna, Austria; 2 The Center for Human Reproduction and The Foundation for Reproductive Medicine, New York, New York, USA; 3 Department of Obstetrics, Gynecology and Reproductive Sciences, Yale University, School of Medicine, New Haven, Connecticut, USA

## Abstract

**Background:**

Impact of fetal gender on prematurity has been primarily investigated in singleton pregnancies. In an attempt to understand better how fetal gender may affect gestational length in twin gestations after in vitro fertilization, same-sex twins and opposite twins were compared for pregnancy duration.

**Methods:**

This study evaluated 113 women at ages 20 to 39 years with consecutive dichorionic-diamniotic twin gestations after assisted reproduction. All pregnancies were results of fresh in vitro fertilization (IVF) cycles with use of autologous oocytes and sperm and were delivered at up to 37 weeks of gestation at a University-based high-risk, maternal-fetal medicine unit.

**Results:**

Both groups did not differ in baseline characteristics, such as maternal ages, indications for fertility treatments, number of previous IVF attempts, body mass index and parity. Opposite sex- twins, however, presented with significantly shorter gestational age at birth (32.9 +/- 3.4 weeks) than same-sex twins (34.3 +/- 2.5 weeks), (p < 0.05). Younger maternal age was also associated with shorter pregnancy duration (p < 0.05).

**Conclusions:**

Fetal gender mix serves as risk factor for more significant prematurity in dichorionic-diamniotic twins after assisted reproduction with opposite sex twins at higher risk than same sex-twins.

## Background

Assisted reproduction considerably contributes to world-wide increases in twin pregnancies in industrialized countries [1,2]. In comparison to singleton pregnancies twin gestations are associated with a significantly higher risk for prematurity and, consequently, prematurity-associated adverse perinatal outcome [3]. To prospectively identify women with twin pregnancies at risk for prematurity is, therefore, crucial. A variety of predictive factors for prematurity risk, such as maternal age, race or obstetric history (such as previous preterm birth) and fetal gender, is well-established according to population-based surveys of mainly spontaneously conceived singleton and twin pregnancies [4-6].

How patients conceive (i.e. naturally or via assisted reproduction) should effect maternal risk factors for preterm birth, like maternal age, race or prior preterm birth [4,5,7]. Mode of conception appears to affect other risk factors, such as fetal gender and chorionicity. For example, Cooperstock et al. described significantly lower prematurity rates in female/female twins when compared to female/male twins [8]. Opposite sex-twins are always dichorionic, while same-sex twins are either monochorionic or dichorionic. Monochorionicity goes along with a significantly higher risk for preterm birth and adverse perinatal outcome than dichorionicity [9].

The lower prematurity rates in opposite-sex twins might, therefore, be at least partly related to chorionicity, as also suggested by Tan et al. [10]. In addition to a potential bias because of chorionicity, the distribution of mono- and dichorionicity differs considerably between spontaneous twins those conceived via assisted reproductive technology (ART). While monochorionicity occurs in as many as one third of naturally conceived twins, it only occurs as rarely as in five percent of ART twins [11]. The impact of fetal gender on prematurity that is seen in spontaneous twins, therefore, does not seem applicable to ART twins.

The largest population-based study on prematurity in twins to date included 3,438 ART and 10,362 naturally conceived twins. It tried to adjust for such confounding factors as chorionicity, parity, maternal age and conception mode. The authors reported similar prematurity rates and somewhat lower perinatal risks for ART twins compared to spontaneously conceived twins, when adjusting for maternal age and parity. The impact of fetal gender and chorionicity on prematurity was, however, only indirectly assessed via sub-analyses of opposite sex twins (i.e. dichorionic twins).

Interestingly, despite lower perinatal overall risk ART twins were at higher risk for admittance to the neonatal intensive care unit (NICU) than spontaneously conceived twins, with even greater disparity when only male/female (F/M) twins were considered [12].

These findings suggest a gender-related impact of perinatal risks in ART twins, with potentially worse perinatal outcome in opposite sex twins after assisted reproduction. They, however, do not allow for differentiation of dichorionic same-sex and dichorionic opposite sex twins on prematurity. To assess the potential effects of fetal sex on prematurity and perinatal outcome in dichorionic twins conceived after assisted reproduction, the present study was initiated. Since twin gestations are to a large majority (i.e. about two third) born preterm [7] and degree of prematurity (i.e. 28 weeks versus 36 weeks) is of significant clinical importance for perinatal outcome, only preterm deliveries were considered for analysis.

## Methods and Results

Out of 218 women with multiple pregnancies after assisted reproduction that underwent prenatal care at the *Department of Obstetrics and Gynecology *at the *Medical University Vienna*, a University-based hospital unit for high-risk maternal-fetal medicine, between July 2002 and June 2008, 113 patients were eligible for enrollment. Inclusion criteria covered exclusively Caucasian women with dichorionic-diamniotic (DC/DA) twin gestations after in vitro fertilization (IVF) with autologous oocytes/sperm in fresh IVF cycles and via the transfer of two or more embryos, who delivered due to spontaneous preterm labor, medical or fetal indications (such as pre-eclampsia, non-reassuring fetal status, intrauterine growth restriction) at up to 37 weeks of gestation [7].

Hansen et al. recently reported higher prematurity rates in ART twins when compared to spontaneously conceived twins [13]. To exclude a potential conception-related bias and to avoid bias due to varying distributions in chorionicity between spontaneous and ART twins, only ART twins were considered for analysis. In Austria, up to four ART cycles per pregnancy are partially covered by a governmental fund, the so-called IVF-Fonds [14]. To qualify for financial support, couples have to be married or in a stable relationship. They have to suffer from one of the following diagnoses, male factor infertility, tubal factor, endometriosis and/or polycystic ovary syndrome and have to be under age 40 (women) or age 50 (men), respectively. About 90% of all fertility centers in Austria offer such financial governmental support and have to report cycle parameters and success rates to a nation-wide registry. More than thirty public and private fertility centers allow for low-threshold to fertility treatment in Austria. Consequently, infertile couples usually exercise their right of financial support. Couples of advanced age, after elective sterilization (i.e., tubal ligation or vasectomy) and with a history of repeated IVF failure generally represent self-pay patients. They are not reported to the nation-wide registry. To ensure a homogenous IVF-population (i.e, diagnosed infertility, limited number of previous IVF attempts, autologous oocytes, female age 18 to 39) and a complete history of IVF-related parameters, such as the number of embryos transferred, only *IVF-Fonds-*covered pregnancies were included. To determine chorionicity, patients underwent first trimester ultrasound scans performed by a small group of experienced, specifically and uniformly trained senior physicians. The detection of lambda signs served as proof of dichorionicity. To exclude potential fetal prematurity-associated biases, neither monochorionic twin gestations, pregnancies with fetal malformations, pregnancies after selective fetal reduction nor pregnancies with vanishing embryos were eligible for enrollment.

To exclude maternal prematurity-associated biases, severe general medical conditions, such as organ transplants or symptomatic autoimmune disease, current or recent cancer treatment, severe disabilities or uterine malformations served as exclusion criteria. Since a number of dichorionic twin gestations were delivered electively after 37 weeks, this could introduce bias into the analysis. This study, therefore, included only IVF twins that delivered up to 37 weeks.

The data analyses of pregnancy- and delivery-related maternal and neonatal outcome data were based on retrospective chart reviews and computer-generated databases at the *Department of Obstetrics and Gynecology *at the *Medical University Vienna*. Assisted reproduction technology (ART)-related data were collected by chart review and from of a computer-generated database at the *IVF-Fonds*. In the study cohort, statistical influences of maternal age, previous IVF pregnancies (i.e., parity and previous preterm birth), body mass index and fetal gender on gestational duration were investigated. Institutional Review Board (IRB) approval for data-linkage and retrospective data analyses was obtained from the IRB at the *Medical University Vienna*. Since our study involved only retrospective chart reviews, informed consent was not required in our patient cohort.

Statistical analyses were performed utilizing SPSS version 10.0. Quantitative variables are summarized by their mean (standard deviation), while qualitative variables are summarized by frequency tables. Univariate and multivariate analyses were performed by linear regression. Patient characteristics known to influence prematurity risk in spontaneous pregnancies (i.e., female age, body mass index, parity and previous preterm birth) [15], as well as fetal gender, were investigated in univariate and multivariate analyses.

Women demonstrated a mean age of 31.6 ± 4.2 years [20-39 years] and a mean body mass index of 23.7 ± 4.3 kg/m^2 ^[17.3-34.3 kg/m^2^]. Considering their infertility history, not surprising, 84.1 percent of all patients had no history of previous IVF-pregnancies. Of those 18 women with a history of a previous IVF-pregnancy, three had a history of preterm birth, one had a history of an intrauterine fetal death with a consecutive preterm stillbirth. Indications for in vitro fertilization treatment were male factor in 52.2%, female infertility, mainly tubal factor, in 28.3% and combined infertility in 19.5%. These results resemble the distributions of infertility diagnoses of all Austrian IVF-Fonds couples [14]. To achieve their dichorionic-diamniotic pregnancy, women underwent a mean number of 1.8 ± 1.2 IVF cycles.

Indications for delivery were preterm labor (with or without preterm rupture of membranes) in 71.7%, fetal indications (such as non-reassuring fetal status or intrauterine growth restriction) in 19.5% and medical indications (such as pre-eclampsia or vaginal bleeding) in 8.8%. The perinatal unit's policy required that pregnancies in premature labor with other than cephalic/cephalic presentations were *universally *delivered by Cesarean sections, while those with two cephalic presentations were *preferably *delivered by Cesarean. This resulted in an overall cesarean section rate of 92.0%.

Delivery occurred at a mean gestational age of 33.8 ± 2.9 weeks and resulted in 112 female and 114 male neonates (i.e., 40 female/male pairs, 36 female/female pairs and 37 male/male pairs). The mean birth weight for both twins was 2053.6 ± 540.6 gm (Twin A) and 1968.8 ± 561.3 gm (Twin B), respectively, with a mean discordance of 9.7 ± 13.1 percent. Mean perinatal arterial pH for both twins was 7.26 ± 0.1 (Twin A) and 7.26 ± 0.1 (Twin B). Delivery resulted in 99.1% live births for Twin A and B. Immediately post partum, 43.4% of first twins and 47.8% of second twins required neonatal intensive care at the neonatal intensive care unit (NICU) of the *Department of Pediatrics *at the *Medical University Vienna*.

Opposite sex twin pregnancies presented with a mean gestational age of 32.9 (3.4 weeks, while same-sex twins delivered at a mean gestational age of 34.3 (2.5 weeks (p < 0.05). No significant differences were observed in maternal age, history of previous IVF pregnancies, maternal body mass index, indications for delivery, birth weight adjusted for gestational age, and mode of delivery between same-sex and opposite sex twins. Neither stimulation protocol used nor cycle characteristics affect pregnancy duration.

When the impact of fetal gender (opposite versus same sex twins) on prematurity, represented by gestational age, was evaluated by univariate regression, a significant association was observed (p < 0.05). Maternal age also reached significance as a predictive factor for prematurity in univariate analysis (p < 0.05). Other factors known to be associated with prematurity in twin pregnancies [16], such as parity, a history of preterm birth, body mass index (BMI) and smoking status, did not show predictive capacity on prematurity risk in univariate analysis (p > 0.05) (Table 1). In multi-regression analysis, including maternal age and fetal sex, maternal age and fetal sex proved significant capacity on prematurity risk, with opposite sex and younger maternal age being significantly associated with shorter gestational duration (p < 0.05) (Table 2). In multi-regression analysis, including fetal gender, maternal age, BMI and previous IVF pregnancy history, fetal gender proved predictive of gestational duration (p < 0.05) (Table 3).

**Table 1:** Univariate analyses on predictive factors of gestational duration in twins after assisted reproduction (n = 113)

	Regression	95% Confidence Interval		p-value
	Coefficient	Lower and Upper Limit		
**Female age (years)**	0,20	0,04	0,29	0,01
**Body mass index (kg/m^2^)**	0,0	-0,01	0,01	n.s.**
**Smoking status**	0,1	-1,31	4,59	n.s.
**Previous IVF pregnancy**	0,1	-0,91	2,08	n.s.
**Indication for assisted reproduction**	-0,1	-0,86	0,74	n.s.
**Stimulation protocol**	0,2	-0,96	2,89	n.s.
**Mode of delivery**	0,10	-0,69	3,30	n.s.
Fetal gender*	-0,20	-2,49	-0,26	0,02

*opposite sex versus same sex twins

**not significant

**Table 2:** Multiregression analysis on predictive factors of gestational duration in twins after assisted reproduction (n = 113)

	Regression	95% Confidence Interval		p-value
	Coefficient	Lower and Upper Limit		
**Female age (years)**	0,2	0,02	0,28	0,02
Fetal gender*	-0,2	-2,33	-0,12	0,03

*opposite sex versus same sex twins

**Table 3:** Multiregression analysis on predictive factors of gestational duration in twins after assisted reproduction (n = 113)

	Regression	95% Confidence Interval		p-value
	Coefficient	Lower and Upper Limit		
**Female age (years)**	0,1	-0,05	0,21	n.s. **
Fetal gender*	-0,2	-2,34	-0,07	0.04
**Body mass index (kg/m2)**	0,0	-0,01	0,01	n.s.
**Previous IVF pregnancy**	0,1	-1,01	1,94	n.s.

*opposite sex versus same sex twins

**not significant

## Discussion

The here presented data for the first time document fetal gender as a risk factor for more significant prematurity in dichorionic twin gestations after IVF, with opposite sex (F/M) twins being at higher risk than same sex-twins. Although underlying mechanisms by which fetal gender impacts prematurity in twins still remain elusive, these data suggest that the determination of fetal gender in twin pregnancies may have clinical value in predicting prematurity risks.

While other studies suggested parity, previous preterm birth and maternal body mass index as risk factors for prematurity [6,16], this study was not able to confirm these findings in ART pregnancies. The lack of influence of parity and previous preterm delivery obviously reflects our infertile patient population of mainly primiparas. Though the number of women with previous (preterm) birth(s) was too small to allow for conclusions, the trends were at the direction one would expect from the existing literature. The same was true for body mass index. Since the majority of women presented with a normal mean body mass index (mean BMI, 23.7 ± 4.3 kg/m^2^), a trend toward higher BMI and lower gestational duration was found in univariate analysis, but did not reach statistical significance.

While opposite sex was confirmed as risk factor for more significant prematurity in all analyses, maternal age also demonstrated a significant association with deliveries at earlier gestational ages. When BMI and obstetric history were also included in the analysis, maternal age, however, lost significance. These results may be age-dependent. In spontaneous twin pregnancies, very young, as well as older prospective mothers of twins are at increased risk for preterm birth [17]. In contrast, multiple pregnancies (with autologous oocytes) after ART usually occur only in younger women. Above age 40 the risk for multiple pregnancies significantly diminishes [2]. Spontaneously conceived twins, therefore, demonstrate a bimodal risk curve with very young and older women at risk for preterm birth, while ART twins demonstrate a linear risk exclusively in young women [18]. This is also consistent with population surveys on the impact of maternal age on ART pregnancies [12,19].

Though we strived for a homogeneous patient population, a limitation of our study is the small sample size of our cohort, compared to register based studies [7]. In a larger sample, the correlation between fetal gender and pregnancy duration should appear more robust. We did not perform cytogenetic evaluation of zygosity. While one could argue that this may have underestimated monozygotic twins, the impact of chorionicity (i.e., dichorionic versus monochorionic) on prematurity risk is well established [9,15,20,21]. Those few studies that have evaluated the effects of zygosity (i.e., dizygotic versus monozygotic) on prematurity risk have, however, so far failed to demonstrate any such association [22,23].

The causative mechanisms for more significant prematurity observed in opposite-sex ART twins remain speculative. Shinwell et al. may, however, have offered an explanation. In a study in very low birth weight twins (500-1500 g) they hypothesized that paracrine signaling that was responsible for higher rates of bronchopulmonary dysplasia and respiratory distress in male twins was also negatively affecting their female co-twins [24]. Higher admittance rates of sex-unlike spontaneous twins to the NICU also point towards gender-mix related risk factors [12]. Even higher NICU admittance for opposite sex twins after ART compared to naturally conceived twins also potentially support paracrine, hormone- and/or immune-modulated effects which allow for adverse effects of males on their female siblings in ART-conceived twins [12].

Though the mechanisms initiating labor remain elusive, one might assume a possible immunological contribution to the onset of premature labor, and, possibly, labor in general [25]. Historically, partial suppression of the maternal immune system during pregnancy was believed to allow the fetal allograft to survive [26]. Recently, in analogy to immunologic tolerance of allogeneic organ transplants, immunologic interactions between fetus (organ transplant) and mother (transplant recipient) have been suggested essential for initiation and maintenance of normal immune tolerance of the mother for her fetus [27].

In further analogy to organ transplantation experiences, it has been also suggested that aberrant immunological responses of mother or fetus can interfere with normal tolerance, resulting in GVHD-like clinical presentations, the most well recognized being preeclampsia/eclampsia [27]. Under the same concept the onset of labor is potentially viewed as the preprogrammed termination of immunologic tolerance of the fetal (semi)allograft at term, inducing uterine activation based on an immunological process akin to an acute GVHD [28]. Premature labor, under such a concept, would, of course, represent the premature termination of maternal tolerance of the fetal (semi)allograft. It, therefore, does not surprise that recipients of organ transplants, doomed to life-long immune-suppression to maintain allograft tolerance, are at substantially increased risk for preterm birth [29].

Maternal immune tolerance of the fetal (semi)allograft appears to involve selected maternal immune activation. The better the maternal immune system is activated, the better maternal tolerance and the more normal will pregnancy proceed [30,31]. Data in the literature strongly suggest that the maternal immune response to the HY protein, encoded by the Y chromosome of the male fetus, enhances maternal immune activation over that achieved by a female fetus. Maternal tolerance is, thus, activated to a higher degree in mothers carrying male fetuses, than in women with female gestations [32]. These observations support the assumption that degrees of immune modulation may play a more important role in pregnancies with male fetal sex than in those with female fetal sex. Such an assumption is also supported by animal experiences. For example, Tai et al reported a significant association between pregnancy duration and degree of maternal immune system activation: The more pronounced the mother's tolerance to the fetus, the more likely the pregnancy was to last [33]. One could, therefore, speculate that twin gestations with fetuses of opposite sex might, consequently, result in difficulties to properly activate maternal tolerance of the fetal (semi)allograft that could finally lead to premature initiation of labor - this, however, remains speculative.

## Conclusions

Our results demonstrate opposite fetal gender as risk factor for more significant prematurity in dichorionic-diamniotic twins after ART, with opposite sex twins at higher risk than same sex-twins. To further elucidate this gender-related impact on prematurity in twin gestations, chorionicity should be included in population based registries.

## Competing interests

The authors declare that they have no competing interests.

## Authors' contributions

AW Substantial contributions to conception and design, acquisition, analysis and interpretation of data, drafting the article, final approval of the version to be published. KK and MSS Substantial contributions to acquisition and contributions to analysis of data, revising the article critically for important intellectual content, final approval of the version to be published. DHB, CW, PH Substantial contributions to conception of the study, analysis of data, revising the manuscript critically for important intellectual content, final approval of the version to be published. NG Substantial contributions to conception and design, analysis and interpretation of data, revising the manuscript critically for important intellectual content, and final approval of the version to be published. All authors read and approved the final manuscript.
